# *NSD2*-epigenomic reprogramming and maintenance of plasma cell phenotype in t(4;14) myeloma

**DOI:** 10.18632/oncotarget.28706

**Published:** 2025-03-21

**Authors:** Andrea Gunnell, Scott T. Kimber, Richard Houlston, Martin Kaiser

**Affiliations:** ^1^Division of Genetics and Epidemiology, The Institute of Cancer Research, London, SW7 3RP, UK; ^2^Present address: Camallergy, Gosport, Hampshire, PO13 0AU, UK

**Keywords:** myeloma, NSD2, t(4;14), CD38, plasma cell

## Abstract

Overexpression of the H3K36 histone methyltransferase NSD2 in t(4;14) multiple myeloma (MM) is an early, oncogenic event, and understanding its impact on genomic organisation and expression is relevant to understanding MM biology.

We performed epigenetic, transcriptional and phenotypic profiling of the t(4;14) KMS11 myeloma cell line and its isogenic translocation knock out (TKO) to characterise the sequelae of NSD2 overexpression.

We found a marked global impact of NSD2 on gene expression and DNA organisation implicating cell identity genes; notably the early lymphocyte regulator, LAIR1 and MM cell surface markers, including CD38, a classical marker of plasma cells which was reduced in TKO cells. Plasma cell transcription factors such as PRDM1, IRF4 and XBP1 were unaffected, suggesting a downstream direct gene effect of NSD2 on cell identity. Changes in cell surface markers suggest an altered surface immunophenotype.

Our findings suggest a role for NSD2 in maintaining MM cell identity, with potential implications for future therapeutic strategies based on targeting of NSD2.

## INTRODUCTION

Multiple myeloma (MM) is a malignancy of plasma cells primarily located within the bone marrow [1]. The disease is characterized by significant molecular heterogeneity and comprises two broad subtypes that reflect different underlying oncogenic pathways of evolution, one characterized by hyperdiploidy and the other by structural changes, most commonly involving translocations of the IGH gene on chromosome 14 [2]. The t(4;14) translocation places *NSD2* under the control of the IgH super enhancer element leading to *NSD2* overexpression in 20% of MM patients who typically have poor survival and do not respond to cytotoxic chemotherapy [3].

*NSD2* is a histone methyl transferase responsible for deposition of H3K36me1 and H3K36me2 marks. Ordinarily, H3K36me2 accumulates on active gene bodies acting as a signature of transcriptional activity. However, with *NSD2* overexpression H3K36me2 spreads from active gene bodies into intergenic regions accompanied by changes in H3K27ac (a feature of regulatory elements) and CTCF binding. Expansion of H3K36me2 domains also drives compartment switching and alterations in intra-TAD interactions. Collectively, these changes have been shown to result in significant alterations in gene expression and oncogene activation [4].

As the primary cancer driver in t(4;14) MM, NSD2 represents a highly attractive therapeutic target *a priori*. Moreover, knock-down of *NSD2* in cell lines leads to reduced cellular adhesion and tumour growth as well as reduced tumour formation in xenograft models [5, 6]. However, targeting NSD2 clinically is likely to impact tumour cell features globally, and characterising the downstream effects of NSD2 in a model system may therefore improve the development of therapeutic strategies.

To provide a more comprehensive understanding of the consequences of *NSD2* over-expression we examined 3D chromosome organisation, gene expression and the cellular phenotype associated with *NSD2* knockdown. As well as highlighting genes and pathways dysregulated by *NSD2* over-expression our findings provide evidence supporting the role of *NSD2* in maintaining plasma cell identify. Collectively our findings may have implications for the development of NSD2-targeting therapeutic interventions.

## RESULTS

### Transcriptional characterisation

**Figure 1:**
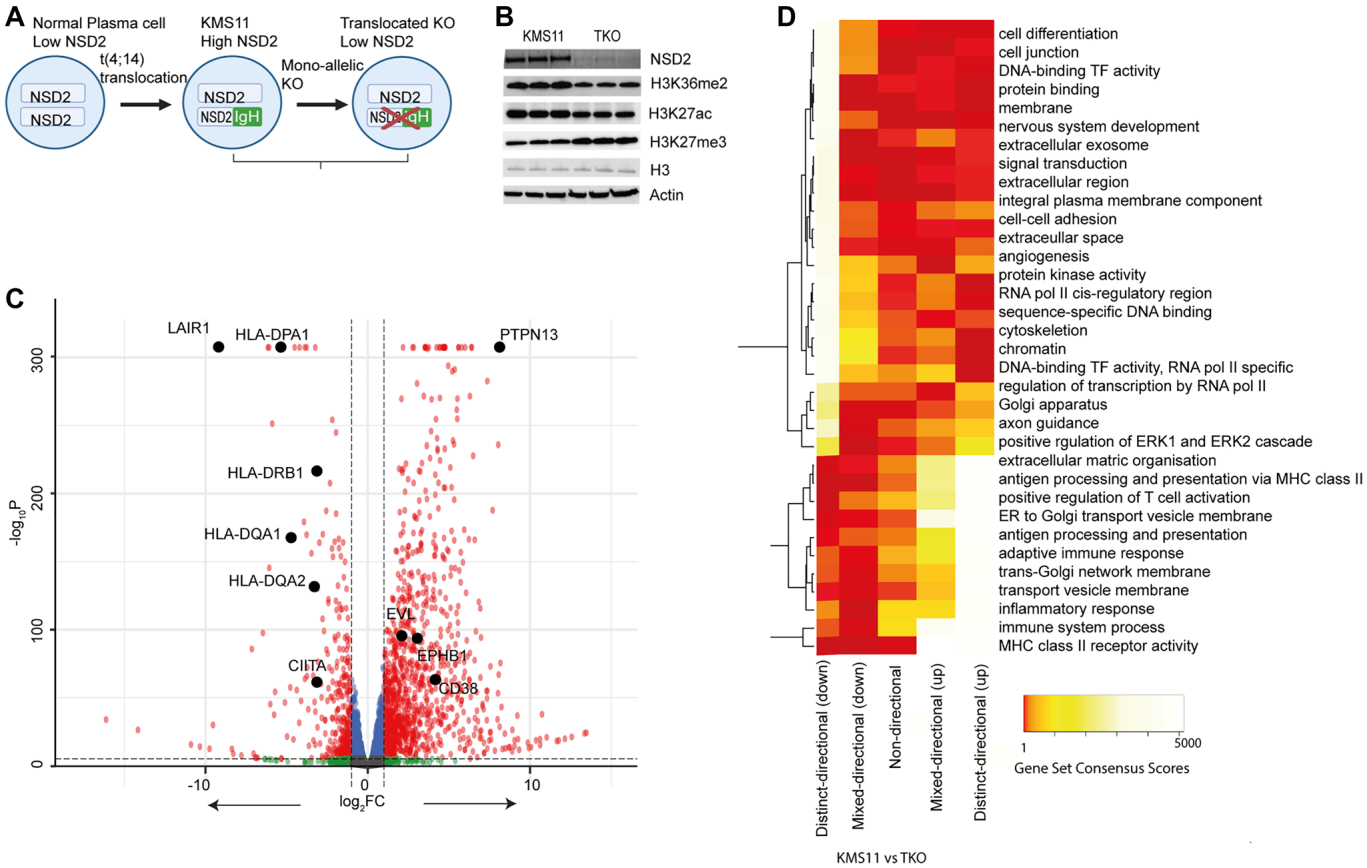
Transcriptional characterisation of KMS11 and TKO cells. (**A**) Schematic of NSD2 status in KMS11 and TKO cells. (**B**) Western blot of histone expression. (**C**) Volcano plot of Log_2_ fold-change in RNA expression from KMS11 to TKO cells is plotted against -log_10_ P. Expression changes of >2-fold and significance *P* < 0.01 thresholds are shown with dotted line. (**D**) Heatmap of Piano analysis gene set consensus scores for KMS11 vs. TKO RNA expression. The consensus score is the mean rank given each gene set by the different GSA runs. A low score (e.g., 1) is a gene set that is ranked high by most of the GSA methods.

To investigate the role of *NSD2* in MM we studied the patient derived t(4;14) cell line KMS11 and its isogenic derivative cell line TKO, in which the translocated allele is inactivated by insertion of a stop codon after exon 6, resulting in truncated NSD2 lacking functional domains (Figure 1A). Hence the two cell lines reflect NSD2 high (i.e., over-expression) and low expression respectively. Overall, the levels of H3K27ac and H3K36me2 were higher in KMS11 cells and levels of H3K27me3 higher in TKO cells (Figure 1B). Based on mRNAseq, NSD2 high expression was shown to be associated with the dysregulation of multiple genes; there were 674 upregulated and 131 downregulated genes (log2 fold change >2, *P* < 0.01; Figure 1C and Supplementary Table 1). The most significantly upregulated gene in KMS11 vs. TKO was *PTPN13*, whereas *LAIR1* was the most downregulated. We performed pathway enrichment analysis of directional gene expression using Piano; NSD2 transcriptional reprogramming was enriched for multiple pathways, including upregulation of cell differentiation pathway genes and down-regulation of the MHC-class II genes (e.g. HLA-DPA1, HLA-DRB1, HLA-DQA1 and HLA-DQA2), which were amongst the most significantly enriched pathways (Figure 1D).

### Genomic organisation

**Figure 2:**
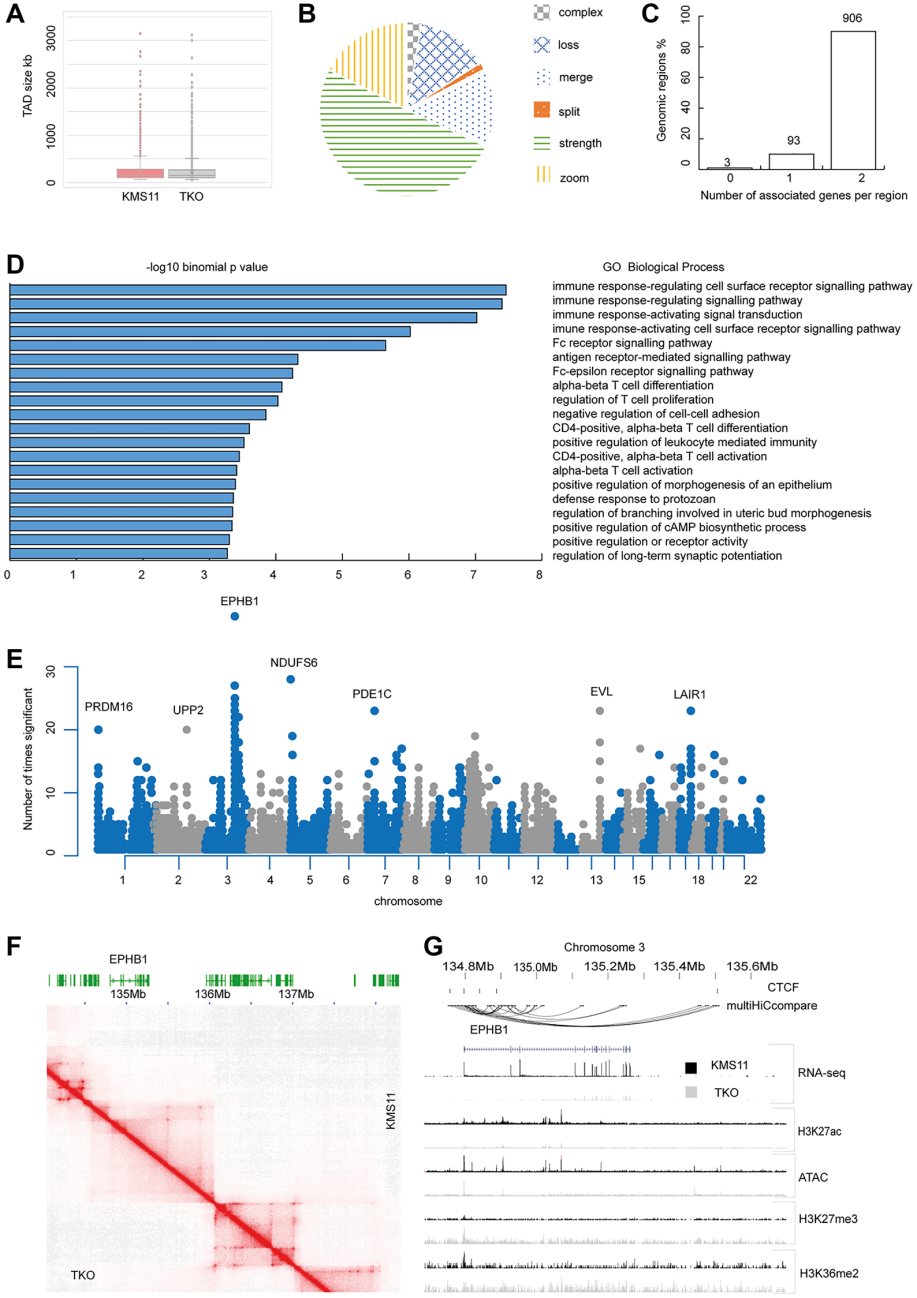
Genomic organisation of KMS11 and TKO cells. (**A**) Box and whisker plot of TAD sizes (**B**) Pie chart of reorganised TAD classification (**C**) Number of genes associated with each TAD (**D**) Gene Ontology Enrichment at reorganised TADs (**E**) Count plot of significant differential interactions. Genes with 20 or more significant interactions are labelled. (**F**) Heatmap of Micro C data at EPHB1 locus in KMS11 vs. TKO cells at 10 kb resolution with balanced normalisation (**G**) Epigenetic landscape at EPHB1 locus in KMS11 and TKO cells showing CTCF sites, differential interactions called by MultiHiCcompare (*p* < 1E-06), RNA-seq normalised counts, scaled H3K27ac, H3K27me3 and H3K36me2 chIP-seq peaks and ATAC-seq peaks.

To examine the effect of NSD2 expression on the organisation of topologically associated domains (TADs) and intra-TAD interactions we profiled KMS-11 and TKO cell lines using Micro-C. By obtaining 4.1 and 3.7 billion reads for KMS11 and TKO cells, respectively, we generated chromatin interaction maps at 1.5 Kb resolution. 87% of interactions detected were intra-chromosomal, providing for a high cis:trans ratio indicative of high-quality data. The number and size of TADs did not substantially differ between KMS11 and TKO (Figure 2A). 1,002 TADs showed evidence of reorganisation between cell lines; 13% of reorganised TADs being lost in TKO cells, ~50% resulting from strength changes at TAD boundaries (Figure 2B).

Assigning genes to reorganised TADs, we examined if this reorganisation impacted preferentially on a specific pathway and/or biological process. Most TADs contained 2 genes (Figure 2C). The genes showing the strongest enrichment were those mapping to pathways mediating immune response; principally, immune response-regulating cell surface receptor signalling pathway (Figure 2D). Using MultiHiCcompare we tested for differential chromatin interactions across the genome between KMS11 and TKO cells (Figure 2E and Supplementary Table 2). The TSS of the gene encoding EPHB1 (Ephrin type-B receptor 1) on chr3:134 795 259 - 135 260 467, displayed the largest number of significant differential interactions from its TSS (*n* = 38). Interactions involving *EPHB1,* which overlapped CTCF sites as well as H3K27ac and ATAC peaks, were present in KMS11 but not in TKO cells (Figure 2F, 2G); in TKO cells the H3K36me2 mark at the TSS is lost and an H3K27me3 mark gained. The change in interaction profile reflected in *EPHB1* expression being 4-fold higher in KMS11 cells (*P*_adj_= 2.07 × 10^−89^). Other notable significant differential interactions included the region chr19:54 350 000–54 359 999, centered on LAIR1 (Leucocyte associated immunoglobulin like receptor). Interactions involving LAIR1 being associated with H3K27ac and ATAC marks peaks present in TKO but not KMS11 cells and LAIR1 expression being 500-fold times higher in TKO than KMS11 cells (Supplementary Figure 1). The cytoskeleton remodelling gene *EVL* is also overexpressed in KMS11 cells at a site of significant differential interactions on chromosome 14 (Supplementary Table 1 and Figure 2E). Generally, genes that were differentially expressed genes between cell lines also showed evidence of being significantly differentially enriched for chromatin interactions (log2FC >2, *P*_adj_ < 0.001). Differentially expressed genes were associated with 1–60 differential chromatin interactions (average, 3.7 differential interactions per gene).

Differential chromatin interactions associated with differential gene expression showed an enrichment for haematopoietic cell lineage genes (hsa 04640), including the cell surface glycoprotein, *CD38*, MHC class II molecules *HLA-DPA1*, *HLA-DPA2*, *ITGA2* and *MME* (enrichment ratio 4.4, *P* = 0.005).

Differential chromatin interactions between KMS11 and TKO also showed an enrichment for T-cell receptor signalling pathways and Natural killer cell mediated cytotoxicity (enrichment 13.7 and 12.7 and *P*-values 0.002 and 0.0009, respectively) including *IFNG*, *PAK1* and *PIK3R1*. *PAK1* and *PIK3R1* also show significant differential expression but *IFNG* does not. *IFNG* does, however, have differential ATAC peaks and looping present in KMS11 that are lost in TKO (Supplementary Figure 2).

### Epigenetic landscape at plasma cell transcription regulators

The plasma cell transcription factors (TFs) *PRDM1*, *IRF4* and *XBP1* were highly expressed in both KMS11 and TKO cells with no evidence of differential expression, epigenetic marks, or chromatin interactions (Supplementary Figures 3–5). *BCL6*, *PAX5* and *EBF1*, which are silent in plasma cells are not expressed in either of the cell lines. However, there were differential chromatin marks and accessibility at each of these loci: H3K36me2 peaks and chromatin interaction loops were markedly lost in TKO at the *BCL6* and *EBF1* loci (Supplementary Figures 6–8). *CIITA*, a master regulator of MHC II expression, which is usually silenced in plasma cells showed increased expression in TKO cells and higher H3K36me2 occupancy (Supplementary Figure 9).

### Cellular phenotype associated with *NSD2* over-expression

**Figure 3:**
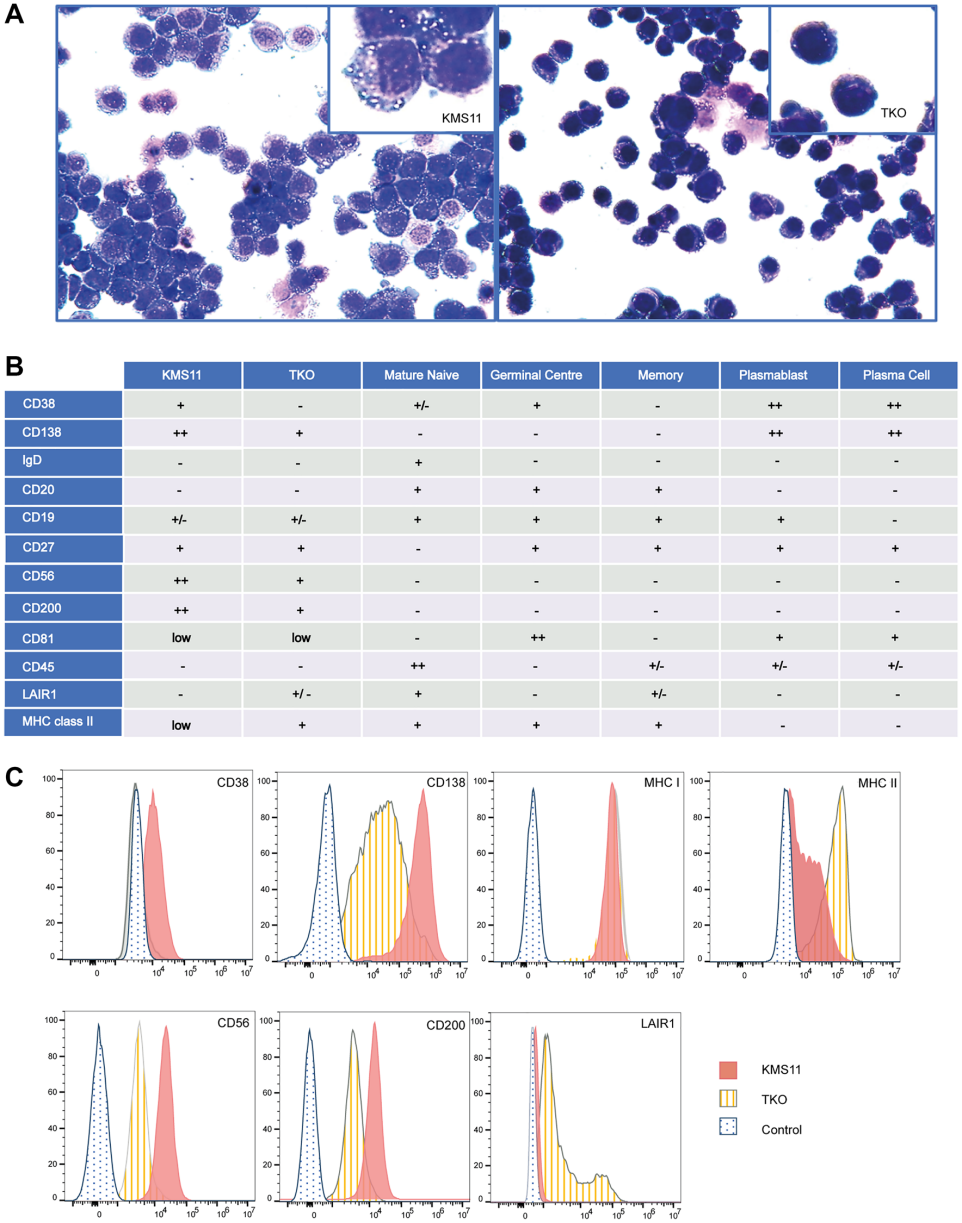
Cellular phenotype associated with NSD2 over-expression. (**A**) Modified wright staining of KMS11 and TKO cells at ×40 magnification (**B**) Summary of surface expression by flow cytometry in KMS11 and TKO cells compared with published B-cell sub-type expression + expressed, ++ highly expressed, -expression not detected, +/− expression in a sub-population, low signal just above background (**C**) Flow cytometry mean fluorescence intensity (x-axis) vs. modal counts (y-axis) for CD38, CD138, HLA-A/B/C (MHC1), HLA-DR (MHCII), CD56, CD200 and LAIR1 antibody staining.

To explore the phenotypic consequences of *NSD2* over-expression we first compared the cellular morphology of KMS11 and TKO cells. KMS11 cells were larger, with eccentric nuclei, coarse chromatin, and abundant cytoplasm; akin to classical plasma cells. In contrast, TKO cells tend to be smaller, with scant cytoplasm (Figure 3A). While KMS11 cells were semi-adherent in culture, TKO cells grew fully in suspension, suggesting cell surface interaction changes. As previously documented, knock out of NSD2 in KMS11 cells is not lethal and the proliferation rate of TKO cells in liquid culture is not substantially reduced. However, colony formation of TKO cells is reduced in methylcellulose [6]. We profiled common MM cell surface markers using a multi-colour flow cytometry panel (Figure 3B). While CD138 (SDC1), CD56 (NCAM1), CD38 and CD200 were all highly expressed in KMS11 cells, all four markers showed lower expression in TKO cells. Using flow cytometry, we also confirmed differential surface protein expression of LAIR1 and MHC II molecules that corresponded with chromatin looping and transcriptional differences (Figure 3C).

## DISCUSSION

To characterise the consequences of the chromatin modifier NSD2 over-expression in t(4;14) MM on genome organisation we analysed high-resolution chromatin interaction profiles and gene expression data of the isogenic KMS-11/TKO MM cell line pair, producing high and low levels of NSD2. Consistent with previous reports NSD2 over-expression was associated with higher H3K36me2, and H3K27ac occupancy, accompanied by reduced H3K27me3 [4]. NSD2 overexpression was linked with substantial changes in chromatin conformation affecting TAD boundaries and chromatin interactions, which also lead to significant changes in gene expression. While our study would benefit from independent validation using and independent cell line our findings are consistent with the role of NSD2 in maintaining cell identity through deposition of H3K36me2 at key genes and the concept of primed genes with increased accessibility prior to increased transcription.

By analysing differential chromatin interactions, we provide evidence to implicate several specific genes in mediating the effect of NSD2 over-expression via TAD reorganisation in MM. Notably, these include EPHB1 whose expression promotes differentiation of dendritic cells [7] and has been linked to MM proliferation [8]; EVL, which determines haematopoietic cell fate [9] and the immune inhibitory receptor, LAIR1, which is expressed early in B-cell differentiation but lost in mature plasma cells. The heterogenous expression of *LAIR1* in TKO cells mirrors its expression in memory B-cells [10, 11]. Other genes whose expression was significantly affected by differential chromatin interactions are those relevant to defining haematopoietic cell lineage, including CD38 and MHC II genes. The reduction in CD38, together with expression of MHC II and LAIR1 in TKO cells, suggests NSD2 expression is supporting maintenance of a plasma cell phenotype, a finding consistent with previous observations. H3K36 methylation has recently been implicated in defining cell identity [12] whereby methylation effectively acts as a place holder, opposing H3K27me3 and TF binding. Intriguingly, expression of MHC genes on MM cells has recently been implicated in the efficacy of anti-BCMA bispecific T-cell engaging (TCE) antibody therapy, as a likely required factor enabling T-cell mediated MM killing [13]. Our results on NSD2 mediated repression of HLA-DR (CD74) expression through DNA reorganisation might be clinically relevant for potential TCE escape in t(4;14) MM, but could also open potential avenues for combination therapy approaches with NSD2 inhibitors. TNFRSF17 (BCMA) itself, the target for TCE therapy in MM, was not altered in expression or DNA looping interactions between KMS11 and TKO.

Our data also suggest that plasma cell TF levels, including key regulators PRDM1, IRF4 and XBP1, remain highly expressed in both KMS11 and TKO cells, and BCL6, PAX5 and EBF1, remain silenced in both. However, CIITA, a master regulator of MHC II expression, which is usually silenced in plasma cells by PRDM1 [14] has increased expression and higher H3K36me2 levels in TKO (Supplementary Figure 9). This suggests NSD2 may be involved in the maintenance of plasma cell expression programs downstream of PRDM1, potentially altering transcriptional control at effector gene level, and enabling potentially differential interaction of the cell with its environment.

In our analysis differential chromatin interactions were not always accompanied by significant changes in gene expression. Increased accessibility prior to increased expression has been demonstrated by Scharer et al in the epigenetics B-cell differentiation and T-cell interactions [15, 16]. Although speculative, such a mechanism may apply to the genes mediating T-cell receptor signalling and NK-mediated cytotoxicity such as *IFNG*, the chromatin landscape of which is changed by NSD2 overexpression.

The observation of CD200 and CD56 expression in KMS11 but not in TKO cells is biologically relevant given their expression is restricted to MM cells rather than healthy plasma cells [17–19]. Moreover, it is especially noteworthy that while the CD200 receptor showed evidence of being expressed at different levels by flow cytometry, support for differential expression was supported by the interaction data but not in the RNA-seq data. This finding is consistent with the observation that mRNA for cell surface receptors is frequently expressed at low levels [20].

Identifying the biological consequences of NSD2 over-expression in MM is not only relevant to informing new therapeutic interventions through indirect targeting of downstream effectors, but also to anticipate possible consequences of targeting NSD2 directly. While inhibiting NSD2 therapeutically for t(4;14) MM has so far proven to be challenging, novel small molecule inhibitors have now started entering early phase clinical trials [21]. Loss of NSD2 associated with epigenetic rewiring appears to alter cell identity and surface expression, which has implications for any future therapeutic strategies and avoidance of emergent resistance. Although the relative reduction of CD38 expression linked with low NSD2 could constitute a concern for anti-CD38 monoclonal antibody targeting, the higher expression of MHC II genes could improve immune effector based TCE approaches to t(4;14) myeloma in context of future NSD2 targeting therapy.

## MATERIALS AND METHODS

### Cell lines

KMS11 parental and *NSD2* translocation knockout (KO) cell lines (TKO) were obtained from Horizon Discovery Ltd. (Cambridge, UK, HD108-002). TKO cells contain a stop codon just after exon 6 of the translocated allele resulting in a truncated protein lacking all functional domains [6]. Cells were maintained in culture as advocated; briefly, cells were grown in RPMI-1640 supplemented with L-Glutamine and 10% foetal bovine serum, sub-cultured twice weekly by dilution and tested regularly for mycoplasma infection. Triplicate samples were biological replicates (i.e., 3 different passages of same cell line).

### Western blot

Cells were pelleted, washed twice in ice cold PBS, and lysed in RIPA buffer plus protease inhibitors or with the Abcam Histone Extraction kit (Abcam, ab221031). Lysates were separated on 4–12% SDS PAGE gels and transferred to nitrocellulose membranes. Membranes were blocked prior to incubation with primary antibody (NSD2, actin, H3K27ac, H3K36me2, H3K27me3 and H3 (Abcam ab75359 and ab8224, CST 8173, 2901S, 9733, 4499T)) overnight at 4°C and washed in TBST prior to incubation with appropriate HRP-labelled secondary antibody (anti-Rabbit-HRP and anti-Mouse-HRP (CST 7074P2 and 7076P2) for 1 hr. at RT. Chemiluminescent signals were detected following addition of ECL western blotting substrate (Pierce).

### MicroC

MicroC of each cell line was performed as per [22, 23], but with the following modifications: Cells were fixed at a density of 10^6^ cells /ml in 3mM disuccinimydyl glutarate (DSG) for 20 mins at RT with rotation, followed by the addition of formaldehyde to a final concentration of 1% and further incubation at RT for 10 mins. The reaction was quenched by the addition of glycine to a final concentration of 660 mM and incubation for 5 mins at RT with rotation.

Fixed cells were digested with MNase (Worthington Biochemical, Lakewood, NJ, USA), which was optimised for each cell line and batch, with incubation for 10 mins at 37°C and shaking at 1,000 rpm. Reactions were quenched by the addition of EGTA to 12.5 mM and heating at 65°C for 10 mins, shaking at 1,000 rpm.

End repair and biotin labelling of 10^6^ MNase-digested cells with 30U of T4 PNK (NEB) at 37°C for 15 mins shaking at 1,000 rpm, followed by addition of 35U Large Klenow Fragment (NEB) and incubation at 37°C for 15 mins shaking at 1,000 rpm and addition of biotin 14-dATP, biotin 11-dCTP (Jena Bioscience, GmbH, Germany, NU-809-BIOX and NU-835-BIO14), dTTP, and dGTP to a final concentration of 66 uM and incubation at 25°C for 45 mins with shaking at 1,000 rpm. The reaction was stopped with the addition of EDTA to 40 mM and heating at 65°C for 20 mins.

Ligation was carried out with 10,000U T4 DNA ligase and incubation at 23°C for 3 hours with shaking at 1,000 rpm. Removal of biotin ends was carried out by incubation with 200U Exonuclease III at 37°C for 10 mins with shaking at 1,000 rpm.

### MicroC data analysis

The Juicer pipeline [24] was used to derive Hi-C maps from FASTQ files and resolution calculated with Juicer calculate_map_resolution.

### Juicer arrowhead was used to call TADs

DiffDomain [25] was used to identify reorganised TADs at 10 kb resolution using Juicer Hi-C contact maps and arrowhead domains as input.

MultiHiCcompare [26] was used to identify differential interactions using the Exact Test function. Two replicates for each of KMS11 and TKO cells were compared at 10 kb resolution following removal of centromeres and blacklisted regions and joint normalisation by the fast-loess method. Significant interactions were filtered with the top directories function with parameters logfc_cutoff = 1, logcpm_cutoff = 1, *p*.adj_cutoff-0.01. The significance of enrichment of differential interactions at differential genes was assessed with the permutation test function using parameters *p*.adj_cutoff = 0.01, logfc_cutoff = 2 and num.perm = 10000 for interactions.

### ATAC-seq and ChIPmentation

ChIPmentation and ATAC-seq were carried out as *per* [27–29], in triplicate, for KMS11 and TKO cells. ChIPmentation of histone marks was based on the following antibodies: H3K27ac (Diagenode C15410196), H3K27me3 (CST 9733), H3K36me2 (Active Motif 61019). Data was processed with Nextflow pipelines nf-core ATAC-seq v1.2.1 and nf-core ChIPseq v1.2.2. Chip-seq data was scaled using ChIPseqSpikeInFree [30]. KMS-11 Transcription Factor CTCF data was obtained from ENCODE 3 (ENCFF649QKE).

### RNA-sequencing

RNA was extracted from three replicates of each cell line using the QIAGEN RNeasy plus kit. Stranded RNA-seq was carried out on ribosomal depleted RNA using NEBNext rRNA depletion kit and NEBNext UltraII directional RNA kit. The sequencing was performed with Illumina NovaSeq (100 cycles paired-end mode). Data processing, quality control, mapping, differential gene expression and piano pathway analysis [31] was carried out using the RNAflow pipeline [32].

### Flow cytometry

One million cells were resuspended in 250 ul staining buffer (Biorad) and incubated with fluorescent-labelled antibodies at recommended concentrations for 15 minutes at room temperature (RT) in the dark. After washing in staining buffer cells were analysed with a CytoFLEX S flow cytometer (Beckman Coulter). Live, single cell populations were gated using forward scatter (FSC) and side scatter (SSC) parameters and appropriate compensation set for antibody label combinations. Specifically, IgD- and IgG2a-Alexa Fluor^**®**^ 700 (BD Biosciences 561302,5578800), CD20-APC, IgG2b-APC, HLA-DR-FITC, IgG2b_FITC and HLA-class1 (A/B/C)-PE (Abcam ab272272, ab91534, ab1182, ab91368, ab33257), CD305 (LAIR1)-PE (Clone: NKTA255, eBioscience^**™**^), IgG1-PE (Invitrogen GM4993).

DuraClone RE PC antibody cocktail (Beckman Coulter B80394) was used to detect CD81, CD27, CD19, CD200, CD138, CD56, CD38 and CD45. Cytometry plots were generated using Flowjo^**™**^ v10.8.1 (BD LifeSciences).

### Cell morphology

Cytospins of cell lines were prepared and stained with a modified wright stain (Hematek Stain Pak, Siemens Healthcare), using a Siemens Hematek Slide Stainer, and examined by light microscopy.

### Gene enrichment analysis

Pathway analysis of differential TADs was carried out with GREAT version 4.04 [33–35].

Kegg pathway enrichment analysis of differential interactions was carried out with the WebGestalt Over-Representation Analysis tool with whole genome reference list [34].

## SUPPLEMENTARY MATERIALS







## Abbreviations

NSD2: Nuclear receptor binding SET domain protein 2
MM: Multiple Myeloma
TKO: Translocated NSD2 knock-out
TADs: Topologically associated domains
EPHB1: Ephrin type-B receptor 1
LAIR-1: Leucocyte associated immunoglobulin like receptor-1
TF: Transcription factor; DSG: disuccinimidyl glutarate
FSC: Forward scatter
SSC: Side scatter
