# Supplementary material for: NSD2-epigenomic reprogramming and maintenance of plasma cell phenotype in t(4;14) myeloma
Source: Oncotarget. 2025 Mar 21;16:220–9. doi: 10.18632/oncotarget.28706 (PMC11927793; doi:10.18632/oncotarget.28706)
Supplement: Supplementary file 1 [file oncotarget-16-28706-s001.pdf]

NSD2-epigenomic reprogramming and maintenance of plasma cell phenotype in t(4;14) myeloma

SUPPLEMENTARY MATERIALS

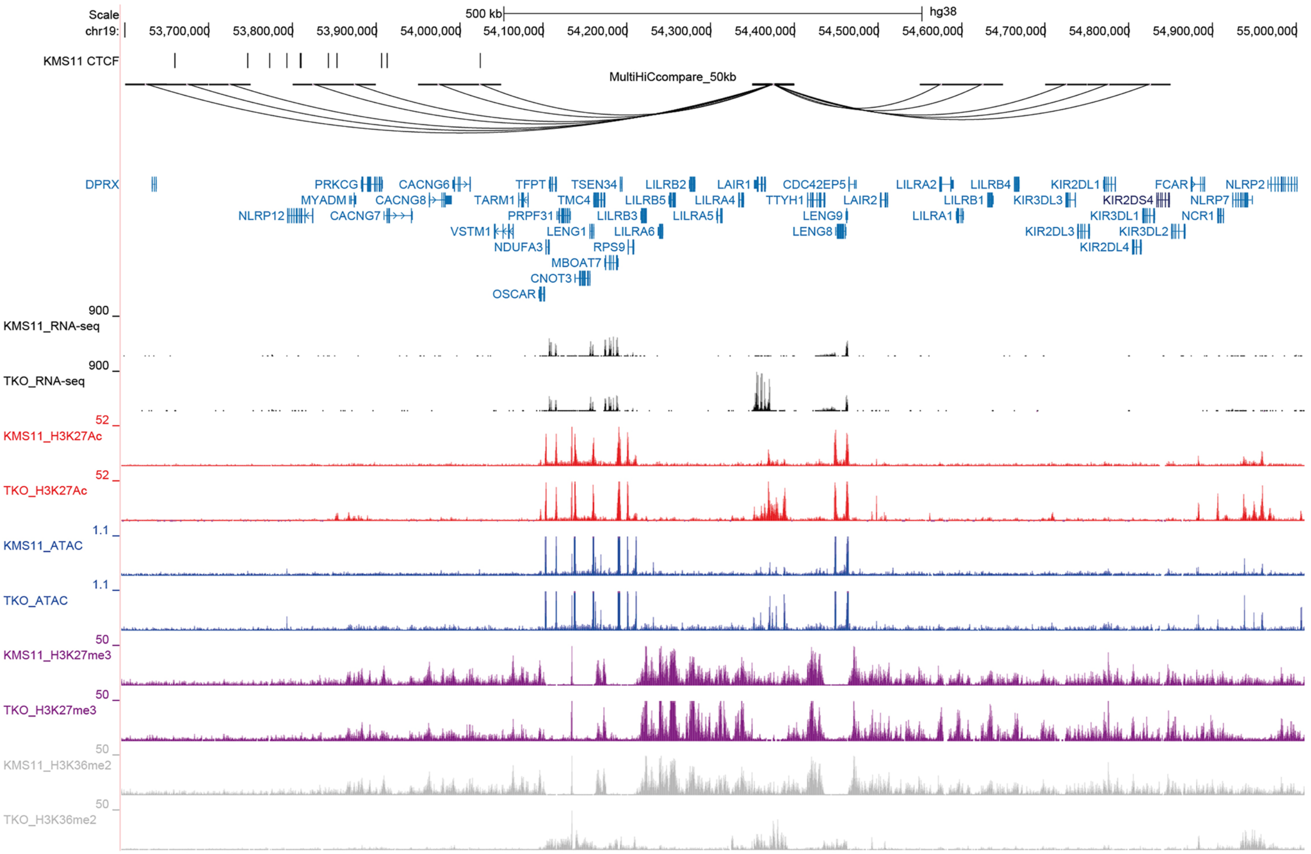

Supplementary Figure 1: Epigenetic landscape in KMS11 and TKO cells at the LAIR1 locus, including KMS11 CTCF sites, differential interactions called by MultiHiCcompare, RNA-seq normalised counts, scaled H3K27ac, H3K27me3 and H3K36me2 chIP-seq peaks and ATAC-seq peaks.

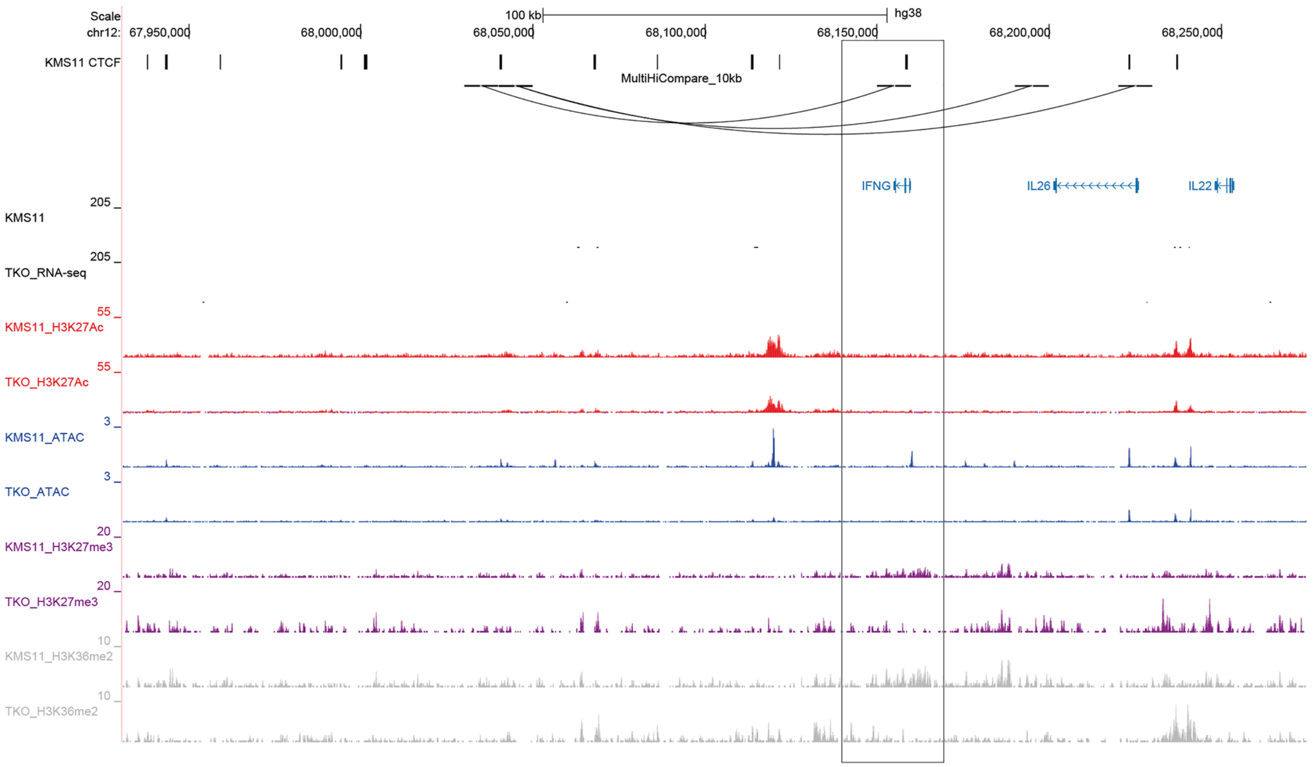

Supplementary Figure 2: Epigenetic landscape in KMS11 and TKO cells at the IFNG locus, including KMS11 CTCF sites, differential interactions called by MultiHiCcompare, RNA-seq normalised counts, scaled H3K27ac, H3K27me3 and H3K36me2 chIP-seq peaks and ATAC-seq peaks.

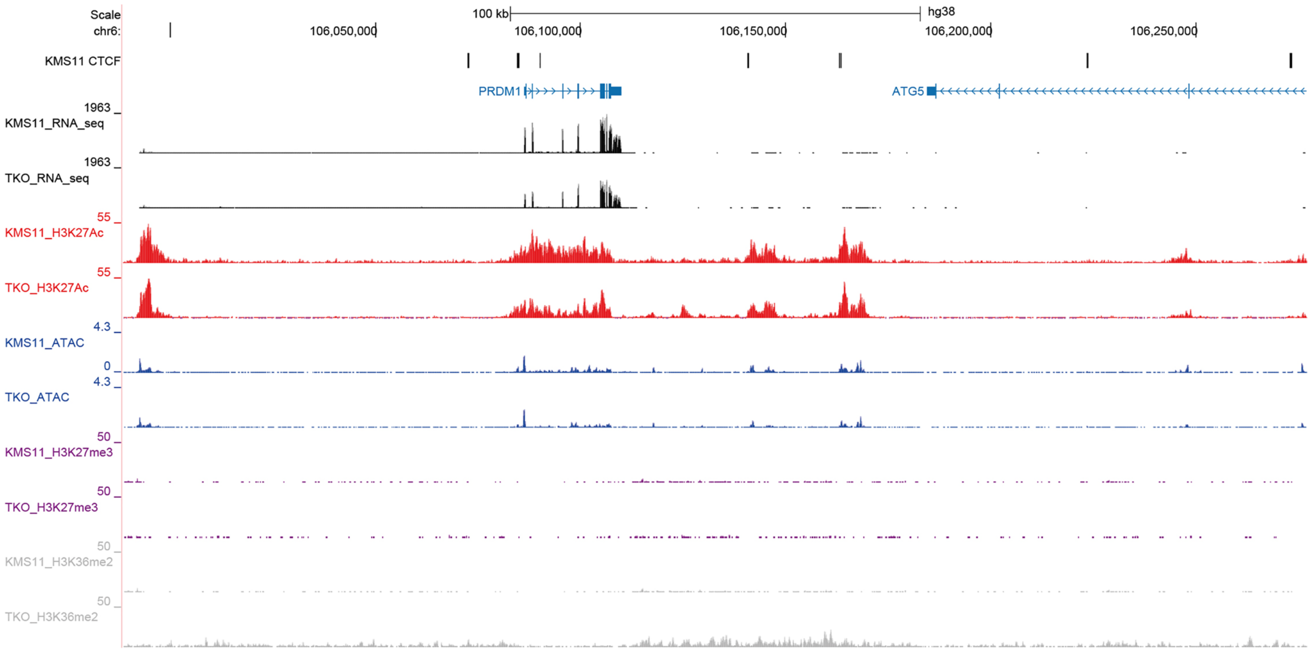

Supplementary Figure 3: Epigenetic landscape in KMS11 and TKO cells at the PRDM1 locus, including KMS11 CTCF sites, RNA-seq normalised counts, scaled H3K27ac, H3K27me3 and H3K36me2 chIP-seq peaks and ATAC-seq peaks. No differential interactions were found by multihiccompare at this locus.

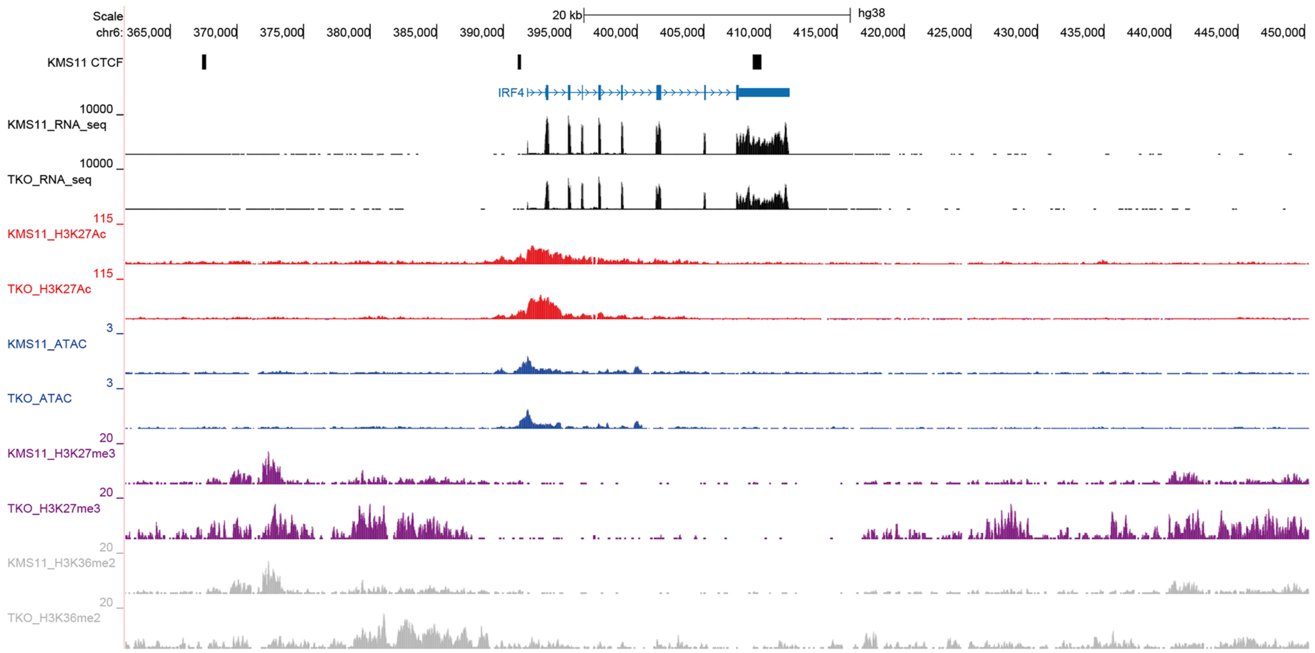

Supplementary Figure 4: Epigenetic landscape in KMS11 and TKO cells at the IRF4 locus, including KMS11 CTCF sites, RNA-seq normalised counts, scaled H3K27ac, H3K27me3 and H3K36me2 chIP-seq peaks and ATAC-seq peaks. No differential interactions were found by multihiccompare at this locus.

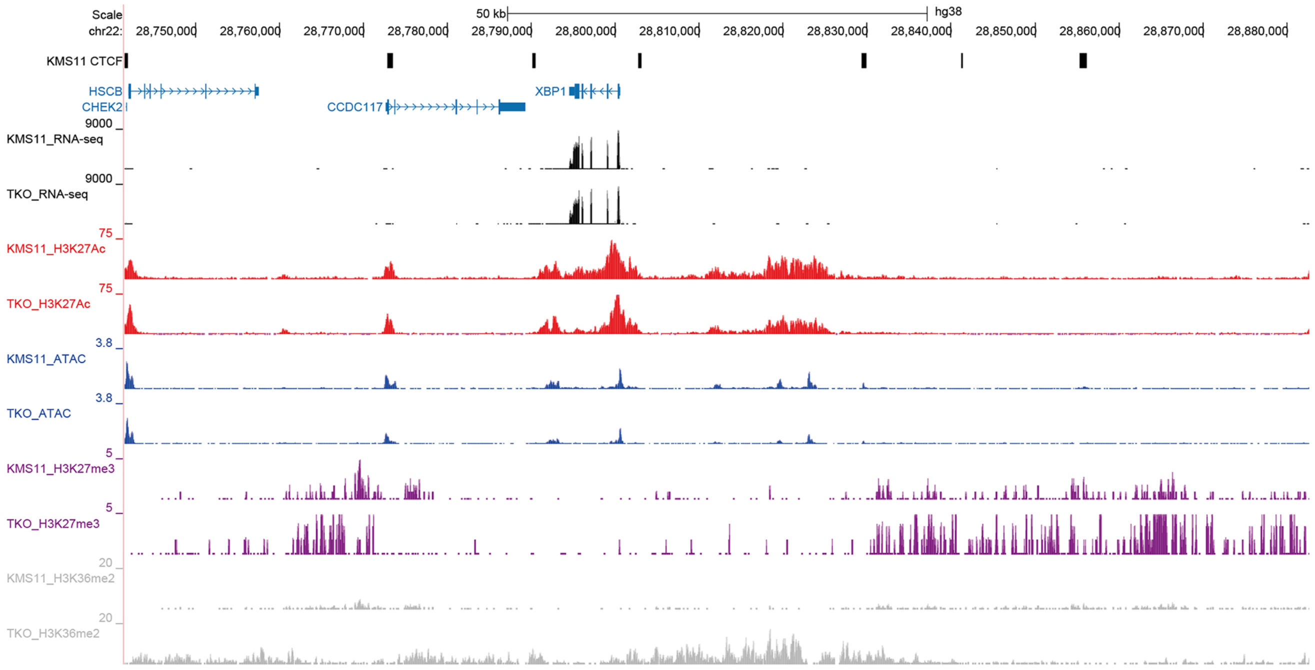

Supplementary Figure 5: Epigenetic landscape in KMS11 and TKO cells at the XBP1 locus, including KMS11 CTCF sites, RNA-seq normalised counts, scaled H3K27ac, H3K27me3 and H3K36me2 chIP-seq peaks and ATAC-seq peaks. No differential interactions were found by multihiccompare at this locus.

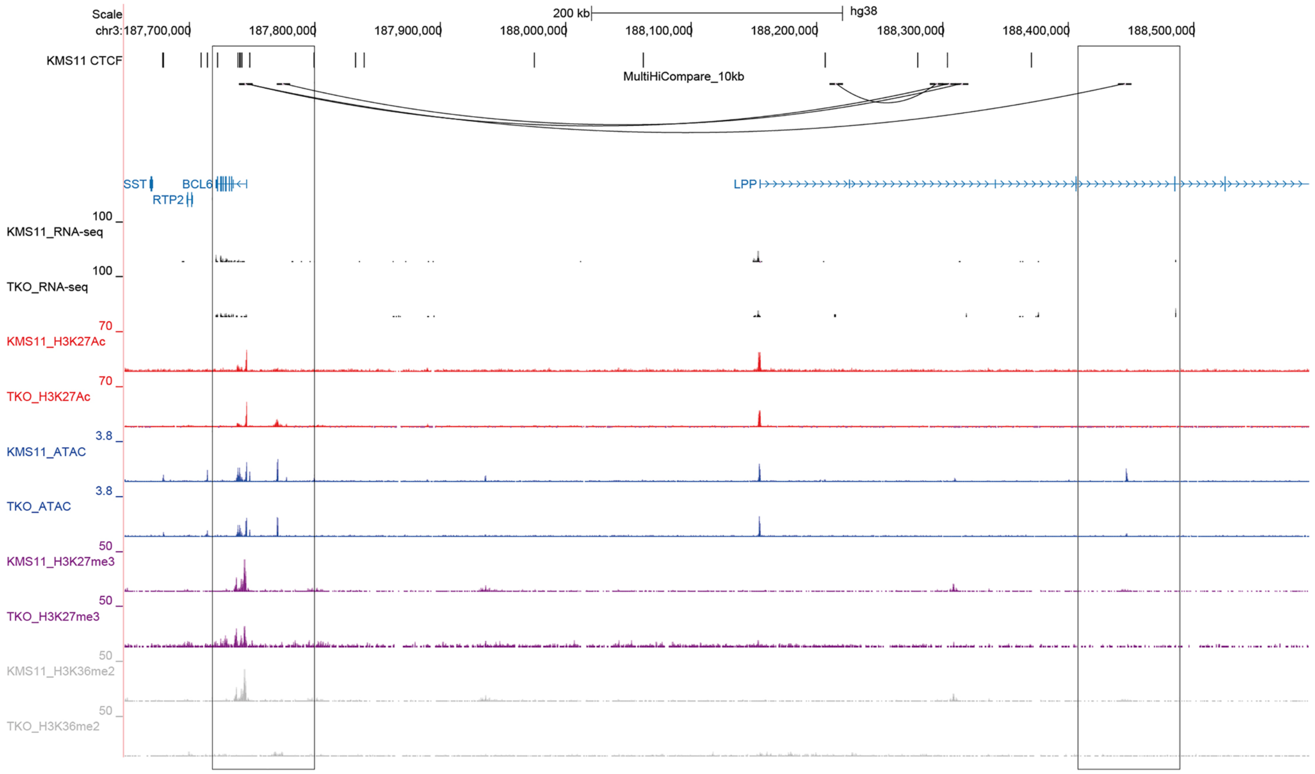

Supplementary Figure 6: Epigenetic landscape in KMS11 and TKO cells at the BCL6 locus, including KMS11 CTCF sites, differential interactions called by MultiHiCcompare, RNA-seq normalised counts, scaled H3K27ac, H3K27me3 and H3K36me2 chIP-seq peaks and ATAC-seq peaks.

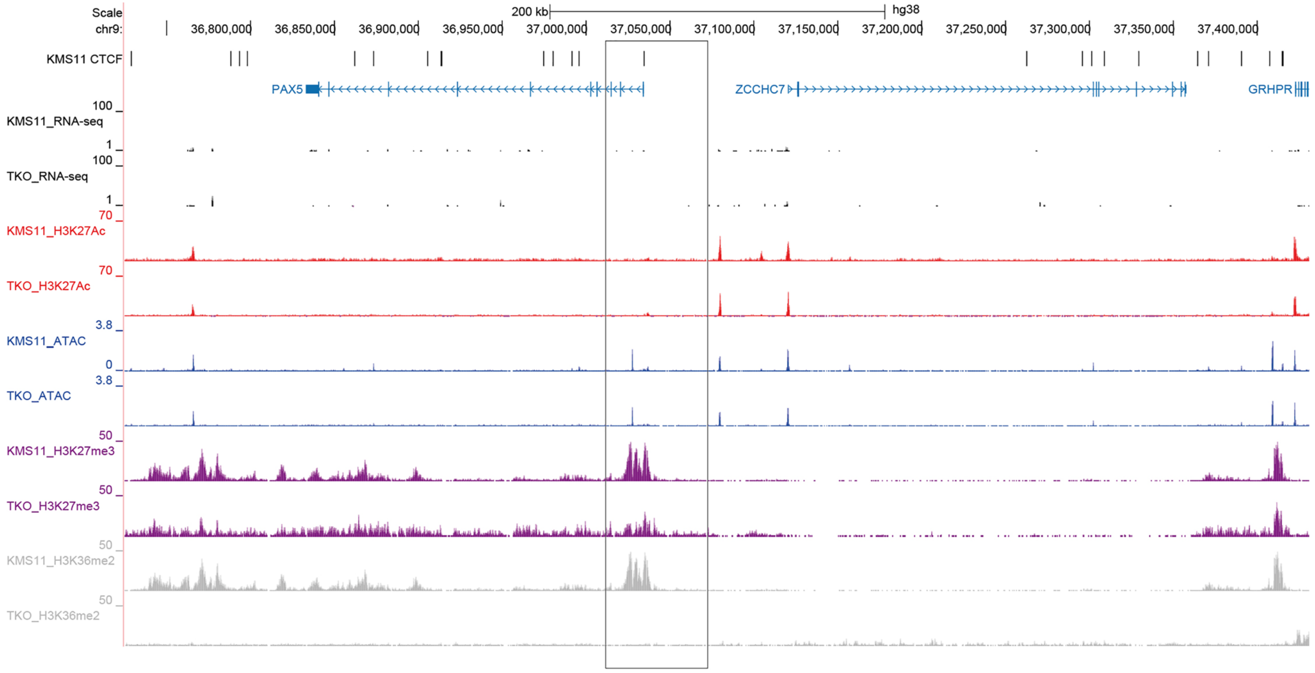

Supplementary Figure 7: Epigenetic landscape in KMS11 and TKO cells at the PAX5 locus, including KMS11 CTCF sites, RNA-seq normalised counts, scaled H3K27ac, H3K27me3 and H3K36me2 chIP-seq peaks and ATAC-seq peaks. No differential interactions were found by multihiccompare at this locus.

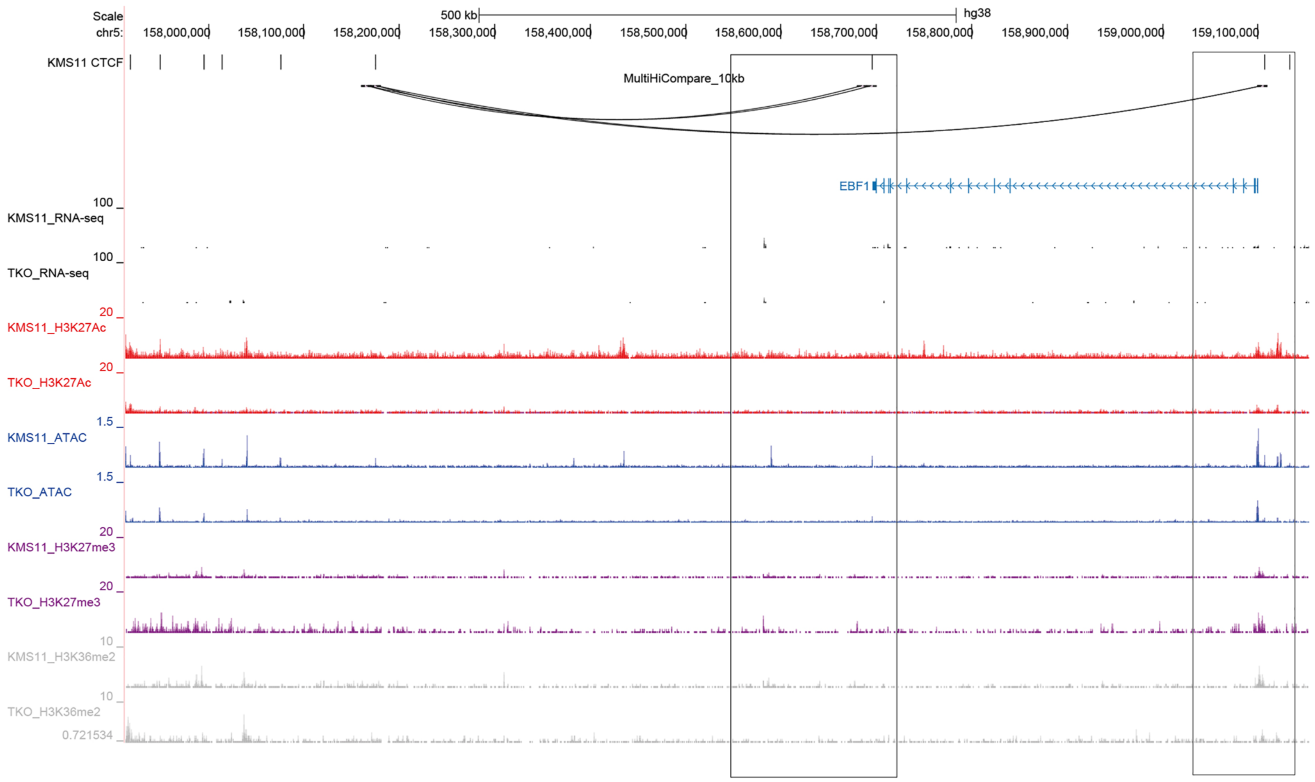

Supplementary Figure 8: Epigenetic landscape in KMS11 and TKO cells at the EBF1 locus, including KMS11 CTCF sites, differential interactions called by MultiHiCcompare, RNA-seq normalised counts, scaled H3K27ac, H3K27me3 and H3K36me2 chIP-seq peaks and ATAC-seq peaks.

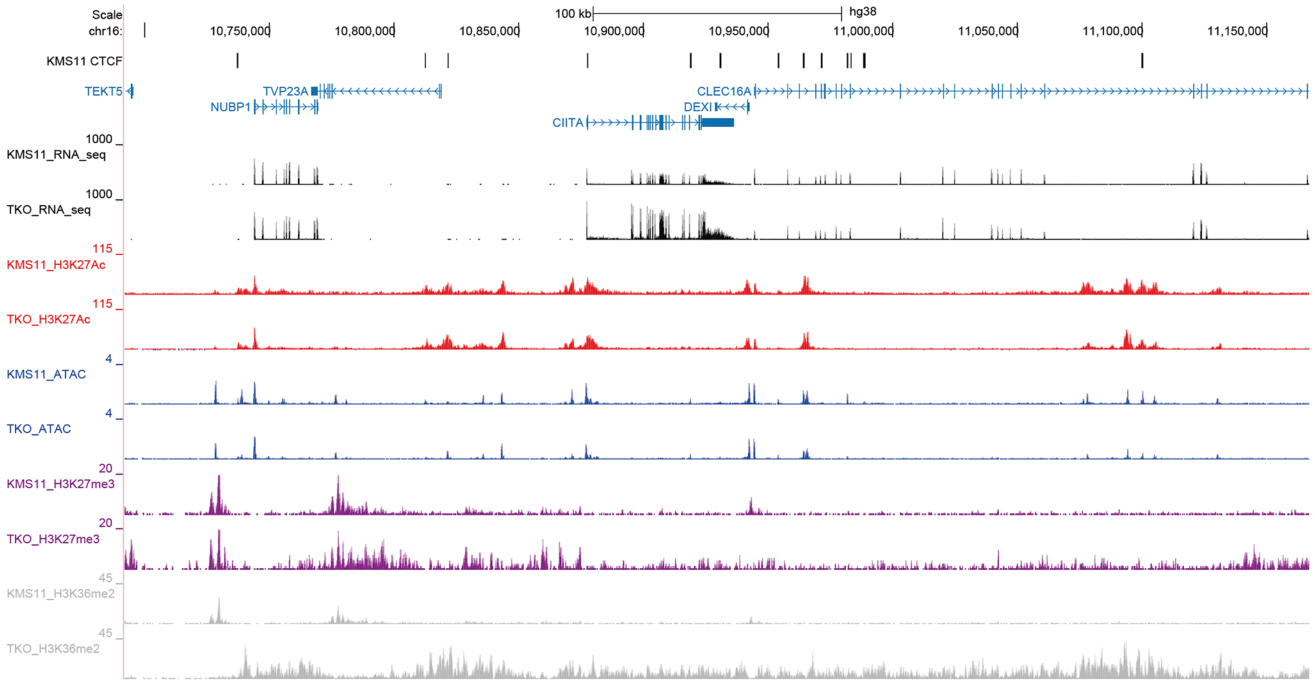

Supplementary Figure 9: Epigenetic landscape in KMS11 and TKO cells at the CIITA locus, including KMS11 CTCF sites, RNA-seq normalised counts, scaled H3K27ac, H3K27me3 and H3K36me2 chIP-seq peaks and ATAC-seq peaks. No differential interactions were found by multihiccompare at this locus.

Supplementary Table 1: RNA-seq analysis results. Results of RNA-seq analysis by RNAflow pipeline. A padj value of 0 is shown where p < 2.225074x 10-308. See Supplementary Table 1.

Supplementary Table 2: MultiHiCcompare results. Results for the MultiHicCompare exact test. D represents interaction distance where 1 = 100 kb. See Supplementary Table 2.
